# Genotypic analysis of clinical and environmental *Cryptococcus neoformans* isolates from Brazil reveals the presence of VNB isolates and a correlation with biological factors

**DOI:** 10.1371/journal.pone.0193237

**Published:** 2018-03-05

**Authors:** Leonardo Euripedes Andrade-Silva, Kennio Ferreira-Paim, Thatiana Bragine Ferreira, Anderson Vilas-Boas, Delio José Mora, Verônica Morais Manzato, Fernanda Machado Fonseca, Kelli Buosi, Juliana Andrade-Silva, Bruno da Silva Prudente, Natalia Evelyn Araujo, Helioswilton Sales-Campos, Marcus Vinicius da Silva, Virmondes Rodrigues Júnior, Wieland Meyer, Mario Léon Silva-Vergara

**Affiliations:** 1 Infectious Disease Department, Triangulo Mineiro Federal University, Uberaba, Brazil; 2 Clinical Pathology Department, Triangulo Mineiro Federal University, Uberaba, Brazil; 3 Molecular Mycology Research Laboratory, Centre for Infectious Diseases and Microbiology, Marie Bashir Institute for Emerging Infectious Diseases and Biosecurity, Sydney Medical School-Westmead Hospital, The University of Sydney, Westmead Institute for Medical Research, Sydney, Australia; 4 Biomedicine Department, Federal University of Piauí, Parnaíba, Brazil; 5 Laboratory of Immunology, Triangulo Mineiro Federal University, Uberaba, Brazil; University of Minnesota, UNITED STATES

## Abstract

Cryptococcal infections are mainly caused by members of the *Cryptococcus neoformans* species complex (molecular types VNI, VNII, VNB, VNIV and the AD hybrid VNIII). PCR of the mating type loci and MLST typing using the ISHAM-MLST consensus scheme were used to evaluate the genetic relationship of 102 (63 clinical and 39 environmental) *C*. *neoformans* isolates from Uberaba, Brazil and to correlate the obtained genotypes with clinical, antifungal susceptibility and virulence factor data. All isolates were mating type alpha. MLST identified 12 known and five new sequence types (ST). Fourteen STs were identified within the VNI isolates, with ST93 (57/102, 56%) and ST77 (19/102, 19%) being the most prevalent. From the nine VNII isolates previously identify by *URA5*-RFLP only four (ST40) were confirmed by MLST. The remaining five grouped within the VNB clade in the phylogenetic analysis corresponding to the sequence type ST504. Other two environmental isolates also grouped within VNB clade with the new sequence type ST527. The four VNII/ST40 isolates were isolated from CSF. The two VNIV sequence types (ST11 and ST160) were isolated from blood cultures. Two of six patients evaluated with more than one isolates had mixed infections. Amongst the VNI isolates 4 populations were identified, which showed differences in their susceptibility profiles, clinical outcome and virulence factors. These results reinforce that ST93 is the most prevalent ST in HIV-infected patients in the Southeastern region of Brazil. The finding of the VNB molecular type amongst environmental Brazilian isolates highlights that this genotype is not restricted to the African continent.

## Introduction

Cryptococcosis causes high mortality in HIV/AIDS patients in limited-resource settings, mainly in sub-Saharan Africa, with a 70% mortality rate being described [1]. In Brazil, where antiretroviral therapy (ART) is provided free of charge by the public health service, mortality rates of 42–60% are still being reported [2,3]. Its clinical presentation commonly is a sub-acute and severe meningoencephalitis prone to dissemination and poor outcome in most cases. These facts are related to the advanced immunodeficiency, delayed diagnosis and sub-optimal antifungal therapy, among others [4,5].

Members of the *Cryptococcus neoformans* species complex account for most cases of cryptococcosis associated with AIDS. Using PCR and sequencing-based techniques four major molecular types can be identified VNI, VNII, VNB, VNIV, in addition to the AD hybrid VNIII. VNI and VNII are worldwide distributed, VNB appears to be endemic to Africa [6], but some reports have suggested a more global distribution [7,8], and VNIV seems to be endemic in Europe [9]. The identification of these molecular types is usually based on the widely used MLST sequencing protocol standardized by the International Society for Human and Animal Mycology (ISHAM). MLST permits to establish better comparisons of the results obtained around the world and favors a evolutionary relationship analysis [10]. Using this scheme, a high variability of isolates from some geographical areas from the African continent compared to other regions highlighted the relevance to characterize isolates of *C*. *neoformans* from different sources and geographical locations in order to outline the global distribution of this species [11–13].

Several studies have compared genetic and phenotypic data of *C*. *neoformans* isolates, however, data correlation between the clinical presentation of cryptococcosis and genotypes of the causative *C*. *neoformans* agents are scarce [11,14]. Considering that the infection is acquired from the environment and not horizontally transmitted among patients, long-term natural selection does not occur by human antimicrobial defence and virulence factors are most likely generated through a natural variation or genetic polymorphism [11,14,15].

The herein reported study aimed to evaluate clinical and environmental *C*. *neoformans* isolates from the Southeastern region of Brazil in order to determine their genotypes and to investigate possible relations between the identified genotypes and the origin, phenotypic characteristics, and antifungal susceptibility of the strains.

## Methods

### Identification and fungal strains

Among clinical and environmental *C*. *neoformans* isolates obtained from 1998 to 2014, 102 were selected for this study (S1 Table). Of these, 63 were recovered from 57 AIDS patients with cryptococcosis admitted at the teaching Hospital of the Triangulo Mineiro Federal University in Uberaba, Minas Gerais State, Brazil. The isolates were recovered from the following respective body sites: 41 from cerebrospinal fluid (CSF), 13 from blood, five from urine, one from bronchoalveolar lavage (BAL) fluid, one from sputum, and two from fragments of skin culture. The remaining 39 isolates were recovered from soil samples and bird excreta from hospital neighbourhood areas and from excreta of captive birds raised in pet-shops distributed all over the city. All isolates were stored at -20°C in 70% Yeast Peptone Dextrose (YPD) broth with 30% glycerol in 2-mL Eppendorf tubes at the Mycology Laboratory for further analyses.

### Sequence analysis of the restriction sites for the *Hha*I and *Sau*96I enzymes of the *URA5* locus and determination of mating types

Genomic DNA was extracted from yeast cells as previously described [16]. Due to discordant results obtained for isolates identify as VNB by MLST in the present study, previously been identified as VNII by *URA5*-RFLP [17,18], an sequence analysis spanning the restriction sites for the *Hha*I and *Sau*96I enzymes in the *URA5* locus was performed. Representatives of the major molecular types: VNI (H99), VNII (WM626), VNB (IUM_97–4515) and VNIV (WM629) were analysed with the software pDRAW32 (http://www.acaclone.com/). The matting type locus was amplified as previously described [19].

### Multilocus sequence typing (MLST)

Sub-typing and molecular polymorphism analysis was performed according to the ISHAM consensus multi-locus sequence typing scheme for the *C*. *neoformans* and *C*. *gattii* species complexes, including seven unlinked genetic loci: *CAP59*, *GPD1*, *LAC1*, *PLB1*, *SOD1*, *URA5* genes and the IGS1 region [10].

Each PCR product was independently sequenced with the forward and reverse primers of each region using the BigDye terminator 3.1 reagent kit (Applied Biosystems, Foster City, CA, USA) with an automated DNA sequencer (ABI PRISM 3130L Genetic Analyzer, Applied Biosystems, Foster City, CA, USA) following the manufacturer’s instructions.

The sequences were manually edited using the software Chromas-pro v. 1.7.6 available at http://technelysium.com.au/ChromasPro.html. Only nucleotide sequences with a Phred quality score ≥20 were included to limit the possibility of incorporating an incorrect base to ≤ 1 in 100 (99% accuracy). The consensus sequences obtained from the forward and reverse reads were generated in Chromas-pro 1.7.6 software. The allele types (AT) and the combined sequence types (ST) were identified via queries against the *C*. *neoformans* MLST webpage at http://mlst.mycologylab.org/. Novel alleles were submitted to mlst.mycologylab.net for assignment of allele and sequence types. Sequences were aligned using the MEGA v. 6.0 software [20].

### Phylogenetic analysis

The phylogenetic analysis was performed in MEGA 6.0 [20]. Consensus sequences of the isolates and those obtained from (GenBank) were aligned with the Clustal W2 algorithm available at https://www.ebi.ac.uk/Tools/msa/clustalw2/ [21]. The allelic sequences for each isolate were concatenated and the evolutionary relationships, with 1,000 bootstrap replicates, were inferred using the neighbour-joining (NJ), unweighted pair group method with arithmetic mean (UPGMA), and maximum likelihood methods (ML) [20]. Major molecular types were confirmed according to phylogenetic clustering with the reference type strains of each major molecular type, available online.

### Population structure

Haplotype networks were generated from the seven concatenated sequence regions to visualize the differences and diversity among the isolates. The number and diversity of each haplotype was estimated using the software DNAsp v. 5.10 available at http://www.ub.edu/dnasp/ [22]. A phylogenetic network was constructed with the Network v. 4.6 software (Fluxus Technologies Ltd, Suffolk, UK) using the median-joining network algorithm [23].

To confirm the haplotypes obtained by median-joining networks and to compare the origin, antifungal susceptibility, and virulence factors with their allelic profiles, a minimum spanning tree was generated in the PHILOVIZ v.1.0 software using the goeBURST algorithm available at http://goeburst.phyloviz.net/ [24,25]. In this analysis, differences between STs are presented as single locus variant (SLV), double locus variant (DLV), triple locus variant (TLV) and up to 7 locus variant, respectively [24,25].

**Fig 1:**
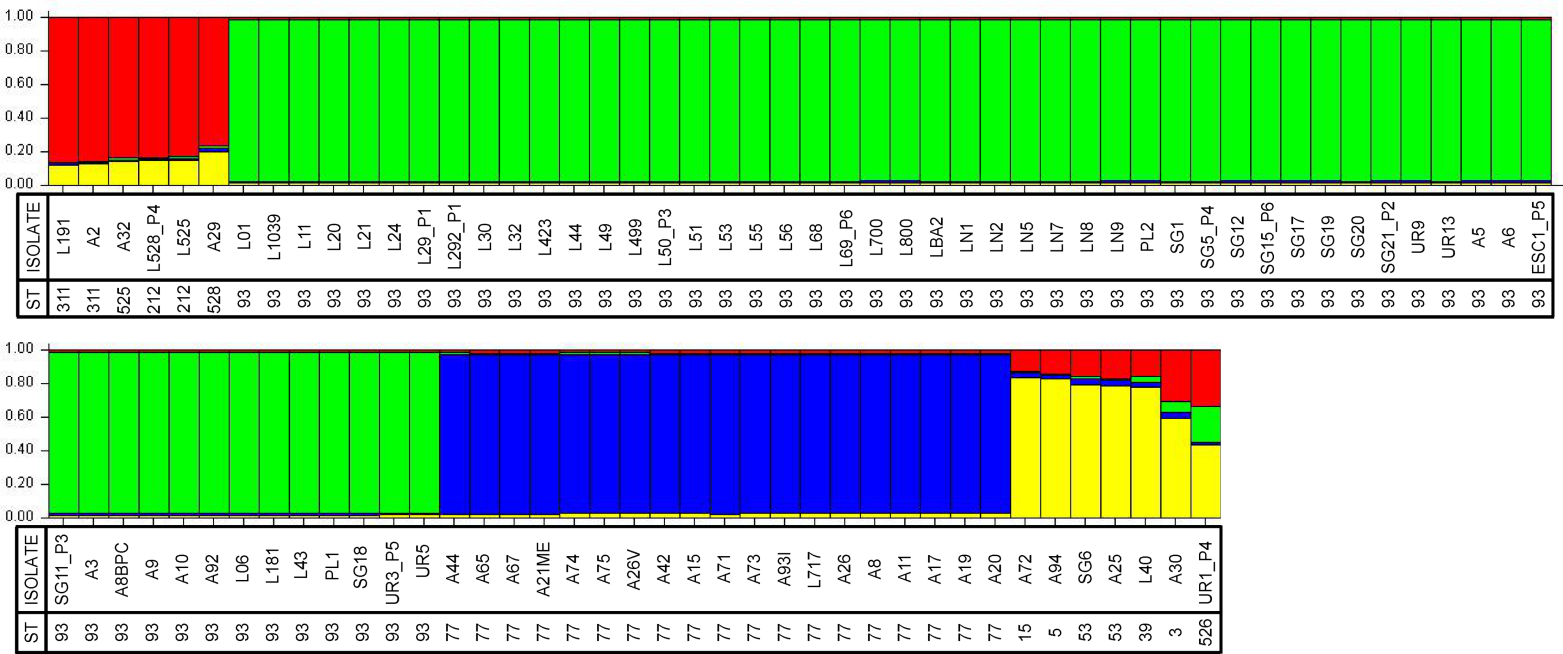
Population structure analysis inferred using sequencing data of the multilocus sequence genotypes of 102 *Cryptococcus neoformans* isolates using K = 4. Legends: ST–sequence type and MT–molecular type.

Analysis in STRUCTURE v. 2.3.4 was performed for the VNI isolates using the admixture model, allowing alpha to be inferred and assuming correlated allele frequencies with a burn-in period of 100,000 Markov chain Monte Carlo (MCMC) replications followed by 100,000 sampling replications. The K number was calculated through the average and standard deviation of each K using the ad-hoc statistic available in Structure Harvester software (http://taylor0.biology.ucla.edu/structureHarvester/) [26]. To evaluate the number of populations only one representative strain of each ST was used. Twenty runs of STRUCTURE analyses were performed for K values 1 to 10, and data were analysed by the Evann and collaborators method as implemented in Structure Harvester [27]. The same conditions in STRUCTURE were applied to the linkage model and the results were compared with those of the admixture model and found to be equivalent. In the figure resulting from this analysis (Fig 1) each vertical line represents one strain with its corresponding sequence type and the different colours represent the most likely ancestry of each individual from the population. Individuals with multiple colours have admixed genotypes from the prior-defined subpopulations.

### Nucleotide diversity

The extent of DNA polymorphisms, such as the number of polymorphic sites (S), nucleotide diversity (p), number of haplotypes (h), haplotype diversity (Hd), and average number of nucleotide differences (k), were calculated using DNAsp 5.10 [22]. In addition, Tajima’s D, Fu & Li’s D*, Fu & Li’s F*, and Fu’s Fs tests for neutrality were calculated. Negative or positive results tests suggest evidence of purifying or balancing selection, respectively. The degree of recombination inside the population was also calculated using the Watterson estimator (theta) method available in DNAsp 5.10. The variance of the estimator decreases with increasing sample size or recombination rate. In addition, the presence of recombination per gene (intragenic), in the concatenated dataset (intergenic) of each population, and in the expanded global dataset was also checked by phylogenetic compatibilities of nearby polymorphic sites along single and concatenated sequences in the software SplitsTree v. 4.13.1 that can be accessed at http://www.splitstree.org/ [28]. Recombination events were visualized by the formation of parallelograms between neighbours using the neighbour-net algorithm [28]. This analysis was calculated applying the uncorrected (observed, "P") distances in characters transformation with the neighbour-net algorithm [28]. The Pairwise Homoplasy Index (PHY) test was used to infer if there was statistical significance for recombination.

## Virulence factors and antifungal susceptibility

The ability of capsular synthesis was measured as previously described [29]. Briefly, 10^6^ CFU/mL of the *C*. *neoformans* isolates were inoculated in Sabouraud broth and in the Sabouraud broth diluted 10 times in MOPS (50 mM pH 7.3), then incubated at 37°C with 5% CO_2_ for 48 h without shaking. After this, the isolates were quantified in a Neubauer's chamber and the size of capsules and yeasts were evaluated in India ink (Trident, São Paulo, Brazil) by light microscopy (Ken-A-Vision, USA) at 1000 times magnification. The reading was made using a micrometric eyepiece (Shimadzu-Kalnew, Japan) calibrated on a micrometer blade (Spencer Lenz Company, USA). The total yeast diameter and total capsule diameter were measured on 20 different cells of each isolate.

Phospholipases, proteases, gelatin hydrolysis, and hemolytic activity were evaluated by the Pz in accordance with previous reports [30–34]. Pz is a quick and simple method to quantify different virulence factors. Pz value is found by dividing the precipitation halo around the colonies by the diameter of the colony. The Pz was interpreted as follows: Pz = 1.0, negative activity; Pz = 0.7 to 0.99, low activity; Pz = 0.5 to 0.69, moderate activity; and Pz <0.5, high activity [35].

The melanin production was evaluated by direct visualization and spectrophotometry measure [36]. The results were reported as optical densities (OD) at 480 nm and represented by the arithmetic mean absorbance values [37,38]. The urease activity was evaluated by spectrophotometry in urea Christensen broth (Difco, USA) [39].

All experiments were performed in triplicate with at least two independent replications. The generated average was used. The reference strains *C*. *neoformans* ATCC 90112 (serotype A), *C*. *neoformans* ATCC 96024 (serotype D) and *C*. *gattii* ATCC 96026 (serotype B) were used as positive controls while *Candida krusei* ATCC 6258 was used as negative control in all experiments.

The antifungal susceptibility tests were performed using the broth microdilution technique following the Clinical and Laboratory Standards Institute (CLSI) recommendations available in the document M27-A3 [40] and its Supplement 4 [41]. Susceptibility to amphotericin B (AMB) (Bristol-Myers Squibb, Princeton, NJ, USA), itraconazole (ITZ) (Janssen Pharmaceuticals, Beerse, Belgium), voriconazole (VRZ) (Pfizer, New York, NY, USA), fluconazole (FLZ) (Pfizer), and ketoconazole (KTZ) (Janssen Pharmaceuticals) was tested.

## Statistical analyses

Statistical analyses of phenotypic and molecular data were performed using Bioestate v. 5.0 available at https://www.mamiraua.org.br/pt-br, MS Excel (Microsoft Corporation) and GraphPad PRISM v. 5.0 available at https://www.graphpad.com. The normality of the data was evaluated using the D’Agostino Pearson test. The homogeneity of variances among groups was tested by Bartlett's test when the data presented normal distribution. After these tests all phenotypic and clinical data analyses were performed by non-parametric tests. The variables were evaluated by the Mann-Whitney test to compare two groups and the Kruskal-Wallis test to three or more groups applying the Dunn's post-test if necessary. The correlation between two variables was evaluated through the Spearman test. To compare the genetic data with categorical variables, the Fisher's exact test or chi-square test were used. P-values less than 5% (p <0.05) were considered statistically significant.

## Ethics statement

All samples of the study were retrieved from the culture collection of Mycology Laboratory of the Triangulo Mineiro Federal University. All data were de-identified. Institutional Human Research Ethics approval for the study was obtained from the Research Ethics Board of the Triangulo Mineiro Federal University (protocols #2711 and #2365). The need for consent was waived by the Ethics Board.

## Results

### Clinical and laboratory analysis

Underlying clinical information was available for 59 patients, the majority of them (57) had AIDS, and two were kidney transplant recipients (S1 and S2 Tables). For statistical analysis, the patients without the respective information on outcome and base disease were excluded. The majority were male (46, 77.9%), with a mean age of 36.5±8.2 years. There was no difference in age between the genders (male = 36.5 ± SE 8.2 vs. female = 34.61 ± SE 12.34, p = 0.53). Among the patients whose isolates were included, 36 (60.0%) had a fatal outcome, regardless of gender (p = 0.46). Within the HIV-infected group with CD4+ T-cell count information available (44 patients), the CD4+ T-cell counts revealed strong association with immunosuppression as 49 (61.3%) of the patients had cell counts below 50 mm^3^/mL. On the other hand, CD4+ T-cell count did not show a statistical association with fatality (p = 0.35).

### Mating type and population structure

All of the 102 isolates analysed presented matting type α. In order to better understand the number of populations and to compare the virulence factors with genotypic data, the admixture model of STRUCTURE identified K = 4 populations in *C*. *neoformans* var. *grubii* (VNI) isolates (S1 Fig, and Fig 1).

The Pop1 marked in red and Pop4 marked in yellow were identified as the most polymorphic populations of this study, and were composed by 4 STs (ST2012, ST311, ST528, and ST525) and 6 STs (ST3, ST5, ST15, ST39, ST53, and ST526), respectively. The Pop1 was composed by three environmental and three clinical isolates and Pop4 by four environmental and three clinical isolates. The major population, marked in green (Pop2), which was composed of 57 isolates, all of the ST93, including seven environmental isolates (A3, A5 A6, A8BPC, A9, A10 and A92). The third population, marked in blue (Pop3), included 19 isolates, all isolates of ST77, 18 environmental isolates and one clinical isolate (L717).

**Fig 2:**
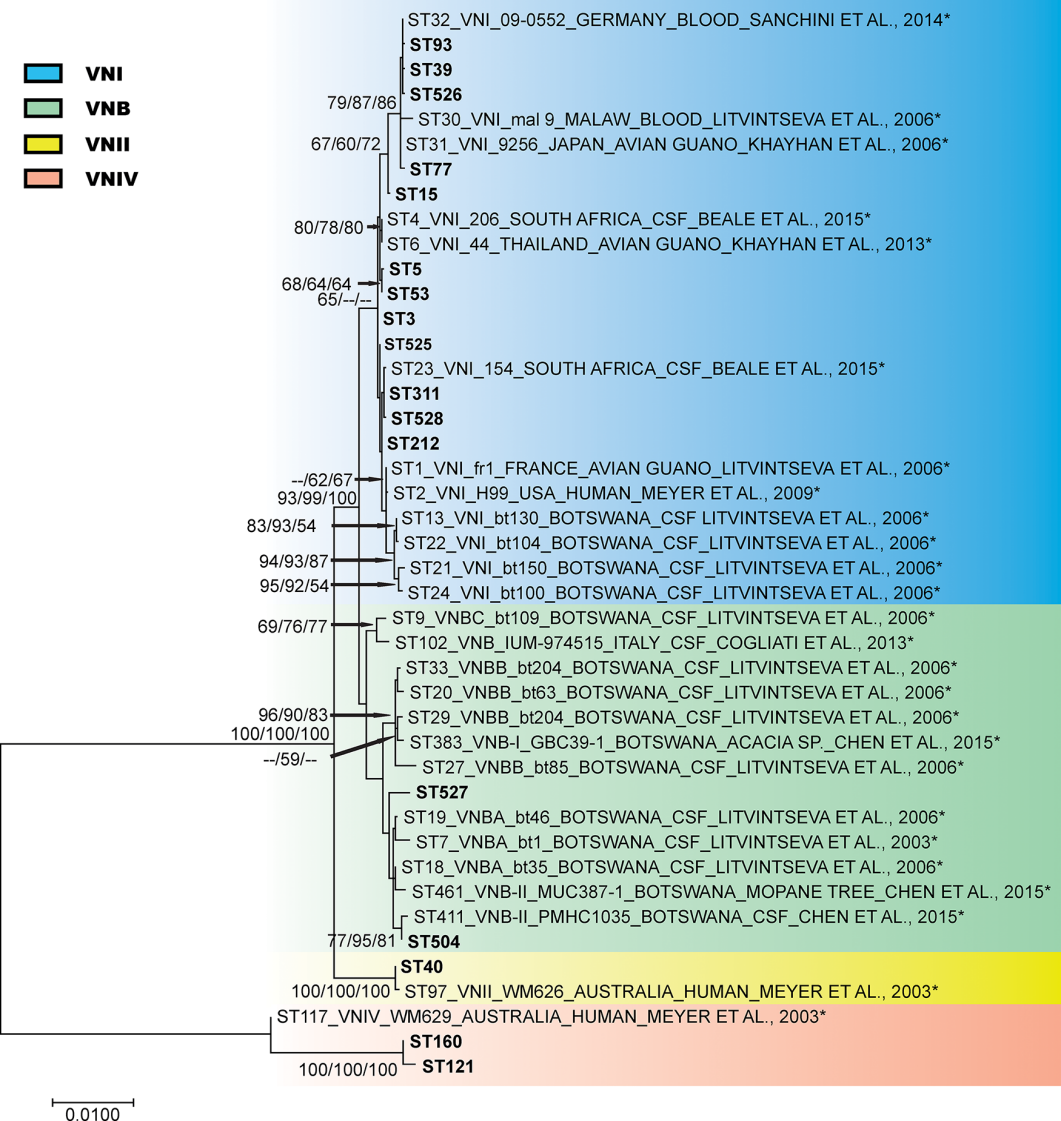
Phylogenetic analysis of Brazilian *Cryptococcus neoformans* isolates inferred by neighbour-joining (NJ), maximum likelihood (ML) and unweighted pair group method with arithmetic mean (UPGMA) methods, using the concatenated data set of the seven MLST loci (*CAP59*, *GPD1*, *LAC1*, *PLB1*, *SOD1*, *URA5*, and the IGS1 region). The analysis involved a total of 43 *C*. *neoformans* isolates, 17 from this study and 26 controls isolates (*) previously published and obtained from MLST database (mlst.mycologylab.org). The phylogenetic tree is drawn to scale, with branch lengths measuring the number of substitutions per site. Codon positions 1st+2nd+3rd+Noncoding were included. There were a total of 4044 positions in the final dataset. Numbers at each branch indicate bootstrap values >50% based on 1,000 replicates by each of the three (ML/NJ/UPGMA) algorithms which presented similar topologies. The isolates identification is described as follows: sequence type number (ST), molecular type, isolate name, followed by isolation source.

The ST527, which comprises two isolates not identified as VNB by the evaluation of the sequences in MLST web site, were grouped within the VNB isolates (isolates A4 and A4M) (Fig 2).

### MLST and genetic variability

A total of 102 isolates were analysed by MLST (S1 and S2 Tables), with five allele types having been identified for the *CAP59* locus, 7 for *GPD1*, 5 for *LAC1*, 6 for *PLB1*, 4 for *SOD1*, 6 for *URA5*, and 9 for the IGS1 region. Based on the combined analysis of the 7 MLST loci, a total of 17 sequence types were observed (Figs 1 and 2; Table 1), with 528 detected polymorphic sites amongst the 4044 sites analysed. The average estimates of these statistics for the concatenated sequences to all isolates reflect a moderate genetic diversity (Hd = 0.653 and π = 0.00633).

**Table 1:** Sources of isolates within the different genotypic groups identified based on the ISHAM-MLST concatenated sequences obtained amongst the 102 *Cryptococcus neoformans* isolates.

Genotypic group (n)			Source (%)							
MT	Pop	ST	Environmental	Clinical	CSF	Blood	Urine	BAL	Sputum	Skin
VNI (89)	Pop1 (6)	ST212 (2)	-	2 (100)	2	-	-	-	-	-
		ST311 (2)	1 (50)	1 (50)	1	-	-	-	-	-
		ST525 (1)	1 (100)	-	-	-	-	-	-	-
		ST528 (1)	1 (100)	-	-	-	-	-	-	-
	Pop2 (57)	ST93 (57)	7 (12.3)	50 (87.7)	32	10	4	1	1	2
	Pop3 (19)	ST77 (19)	18 (94.7)	1 (5.3)	1	-	-	-	-	-
	Pop4 (7)	ST3 (1)	1 (100)	-	-	-	-	-	-	-
		ST5 (1)	1 (100)	-	-	-	-	-	-	-
		ST15 (1)	1 (100)	-	-	-	-	-	-	-
		ST39 (1)	-	1 (100)	1	-	-	-	-	-
		ST53 (2)	1 (50)	1 (50)	-	1	-	-	-	-
		ST526 (1)	-	1 (100)	-	-	1	-	-	-
VNB (7)		ST504 (5)	5 (100)	-	-	-	-	-	-	-
		ST527 (2)	2 (100)	-	-	-	-	-	-	-
VNII (4)		ST40 (4)	-	4 (100)	4	-	-	-	-	-
VNIV (2)		ST121 (1)	-	1 (100)	-	1	-	-	-	-
		ST160 (1)	-	1 (100)	-	1	-	-	-	-

Legend: n–number of isolates; MT–molecular type; ST–sequence type; Pop–populations found in the study using STRUCTURE.

The neutrality tests Tajima’s D, Fu & Li’s D*, Fu & Li’s F*, and Fu’s Fs showed evidence of purifying selection or population expansion for the total isolates, clinical isolates, environmental isolates and Pop1. The highest genetic diversity was evidenced in Pop4 (h = 6, Hd = 0,952 and π = 0.00185), followed by Pop1 (h = 4, Hd = 0,867 and π = 0.00032). The environmental isolates exhibited higher genetic variability than the clinical isolates, with 11STs × 10STs, haplotype diversity of 0.750 × 0.369. The overall genetic diversity of VNI+VNII+VNB was represented by h = 15, Hd = 0,639, and π = 0.00296 (Table 2). The lowest genetic diversity was observed in Pop2 and Pop 3 (h = 1, Hd 0.0 and π = 0.0).

**Table 2:** DNA polymorphisms in different groups and populations analysed based on the ISHAM-MLST concatenated sequences of 102 *Cryptococcus neoformans* isolates studied.

(number of isolates)	Length	S	SVS	PIS	*π*	k	h	Hd	D	FD	FF	FS	Theta-w	Rm
													Vtnr	
													Vnfr	
Total (102)	4044	405	7	398	0.00633	24.817	17	0.653	-2.319	2.37*	0.360	19.48	77.925	7
													360.732	
													14.994	
Clinical (63)	4040	388	9	379	0.00748	29.323	10	0.369	-2.297*	2.033*	0.357	27.755*	82.336	2
													479.57	
													17.472	
Environmental (39)	4001	50	1	49	0.00384	15.347	11	0.750	1.064	1.746*	1.792*	7.699*	11.826	3
													14.180	
													2.797	
VNI+VNII+	4010	84	2	82	0.00296	11.822	15	0.639	-0.8871	2.075*	0.998	7.784*	16.224	6
VNB (100)													18.086	
													3.134	
Pop1 (6)	4004	3	2	1	0.00032	1.27	4	0.867	-0.1854	-0.3748	-0.35156	-1.350	1.3138	0
													0.828	
													0.575	
Pop2 (57)	4003	0	0	0	0.000	0.000	1	0.000	-	-	-	-	0.000	0
													0.000	
													0.000	
Pop3 (19)	4005	0	0	0	0.000	0.000	1	0.000	-	-	-	-	0.000	0
													0.000	
													0.000	
Pop4 (7)	4005	16	4	12	0.00185	7.429	6	0.952	0.7630	0.76214	0.83911	-0.377	6.5306	1
													10.623	
													2.666	

Legend: S–number of polymorphic sites; *π* –nucleotide diversity, Pi; k–average number of nucleotide differences; h–number of haplotypes; Hd–haplotype diversity; D–Tajima’s D; FD–Fu and Li’s D; FF–Fu and Li’s F; Fs–Fu’s Fs

*–p<0,05

Rm–Minimum number of recombination events; Theta w–Theta (per sequence) from S; Vtnr–Variance of theta (no recombination); Vnfr–Variance of theta (free recombination); SVS–Singleton variable sites; PIS–Parsimony informative sites.

**Fig 3:**
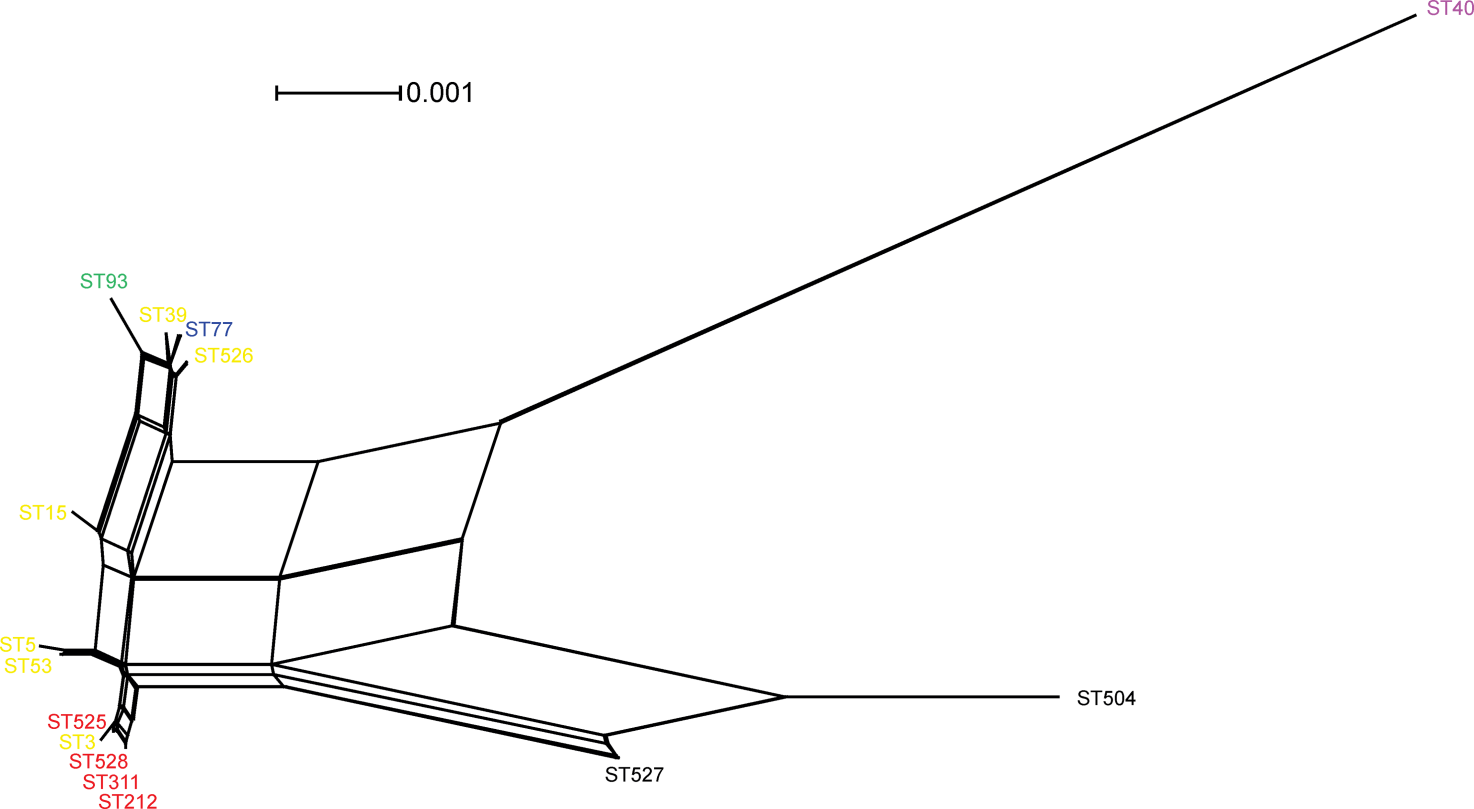
Split decomposition analysis of the concatenated dataset of the 7 MLST loci of the studied *Cryptococcus neoformans* isolates applying the neighbour-net algorithm using the uncorrect-P parameter model and evidencing the diversity and branching ambiguities attributable to recombination events. The observation that isolates are linked to each other by multiple pathways and are forming an interconnected network rather than a single bifurcating tree is suggestive of recombination. The phi test for recombination implemented in the software SplitsTree showed significant evidence (p<0.0001) for recombination. The STs belonging to the main clusters identified in the previous phylogenetic analysis were also separated using the split decomposition and are highlighted as follow: VNII: purple; VNB: black; Pop1: red; Pop2: green; Pop3: blue; and Pop4: yellow.

The presence of recombination was evaluated using the PHI test, the minimal number of recombination events per gene, and the Watterson estimator (theta). The Watterson estimator (theta) method resulted in strengthening the hypothesis of a recombinant population (Table 2). Besides, the estimates of recombination events among the whole *C*. *neoformans* population revealed that at least 7 recombination events had occurred and that the Pop4 was the most recombinant population (1 event). The PHI test performed on the concatenated (intergenic) dataset also showed evidence of recombination (PHI, p*<*0.0001) (Fig 3). Taken together, the results showed evidence for recombination using the concatenated dataset.

Among the analysed isolates, five were identified as VNB, all of them from environmental origins, four from bird excreta within the hospital neighbourhood (BHEN) (A1, A34, A90, and A93II) and one from excreta of captive birds raised in a pet-shop (ECB) (AK11). The five ST504 isolates were identified via the MLST web site, whereas the other two isolates (A4 and A4M) presented a new sequence type ST527 and grouped together with the VNB clade. These isolates were subsequently aligned with isolates of different molecular subtypes and grouped with the isolates of the subtypes VNBA [42], VNBII [43], and VNBII [44] types (S2 Fig). VNBI, VNBII, VNB-I, VNB-II, VNB-A, VNB-B, VNB-C, and VNI/VNB admixture are populations found among VNB isolates from clinical and environmental samples used in studies that included isolates from Botswana [42–44]. The four (3.82%) VNII isolates (ST40) were obtained from CSF whereas the two VNIV (ST11 and ST160) were obtained from blood culture. The VNI isolates were distributed over 14 STs (ST3, ST5, ST15, ST39, ST53, ST77, ST93, ST212, ST311, ST525, ST526, ST527, ST527, and ST528). The most present STs were ST93 (57, 56%) and ST77 (19, 19%). The ST212 and ST311 were composed by two isolates each, whereas the remaining STs had just a single isolate. The ST93 was composed of clinical isolates (50/57, 88%), whereas the ST77 was predominately composed of environmental isolates (18/19, 95%) (Table 1).

Among the environmental isolates most were isolated from bird excreta of the hospital neighbourhood 35 (89.7%), three from soil samples of the hospital neighbourhood (A29, A30, and A32) and one from excreta of captive birds raised in a pet-shop (AK11). Due to the low number of isolates from captive birds (three) and from soil samples (one), more in deep comparisons were not performed. All of these isolates presented different sequence types, being three of them VNI (ST528, ST3, and ST525) and one VNB (ST504).

MLST sequencing allowed for the identification of five VNB isolates, which had been previously genotyped by *URA5*-RFLP as VNII [17,18]. To evaluate this contradiction sequence analysis of the restriction sites with characteristics of the four major genotypes of *C*. *neoformans* was performed (S3 Fig). This analysis showed that VNB and VNII isolates have the restriction site for the *Sau*96I enzyme in the same position and therefore present the same restriction profile by *URA5*-RFLP.

**Fig 4:**
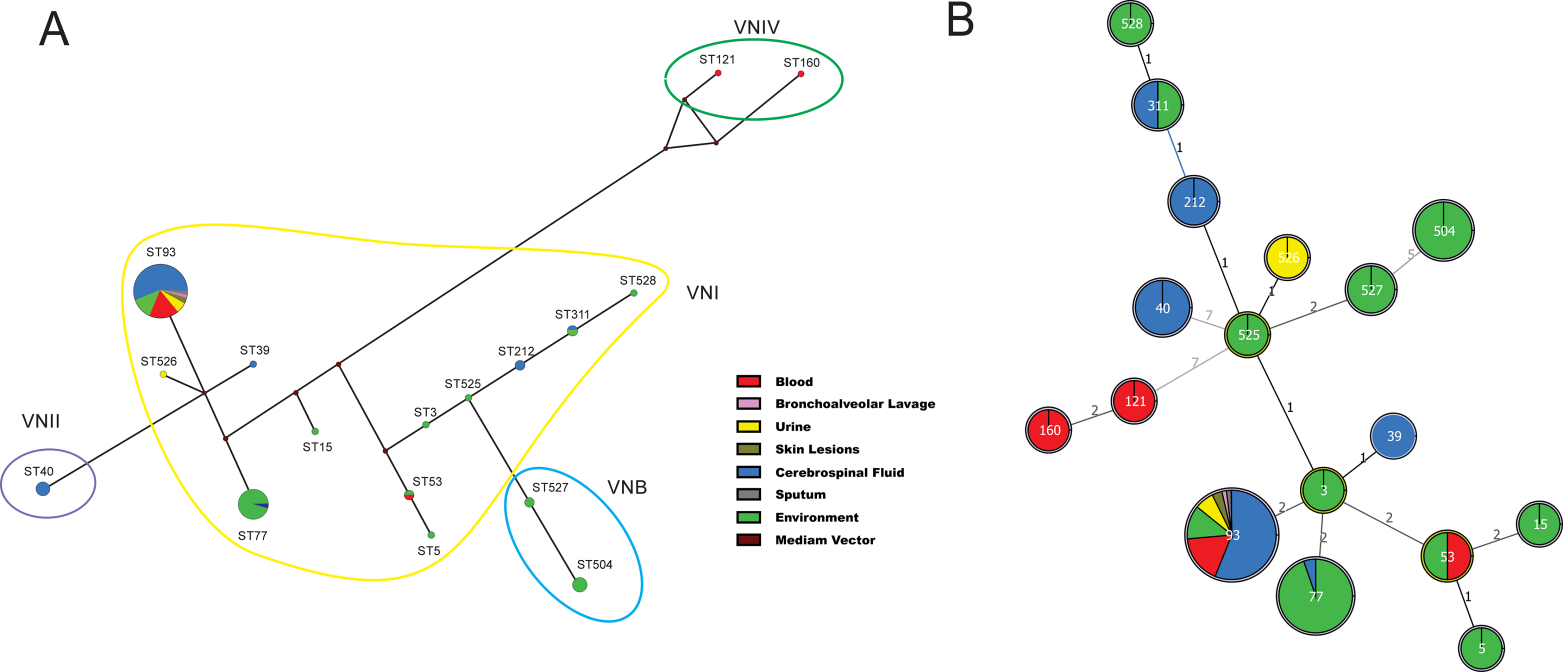
**Median-joining haplotype network (A)** of 102 clinical and environmental *C*. *neoformans* isolates based on concatenated nucleotide sequences of the seven MLST loci. The major molecular types found in this study (VNI, VNII, VNIV, and VNB) are indicated by circles of different colours. Each circle represents a unique Sequence Type (ST), and the circumference is proportional to sequence type frequency (ST93: 77 isolates; ST77: 19; ST504: 5; ST40: 4; ST53: 2; ST527: 2; ST311: 2; ST212: 2; ST528: 1; ST526: 1; ST525: 1; ST160: 1; ST121: 1; ST39: 1; ST15: 1; ST5: 5; ST3: 1). Brown dots (median vectors) are hypothetical missing intermediates. **Minimum spanning trees (B)** using the goeBURST algorithm among *C*. *neoformans* isolates determined by median-joining network analysis. The size of the circle corresponds to the number of isolates within that haplotype, and the numbers between haplotypes represent the genetic distance of each haplotype, excluding the gaps. The figure shows the distribution of sequence types according to the clinical site and environmental origin.

The goeBurst analysis and median-joining haplotype network were applied to infer patterns of evolutionary descent among clusters of related genotypes and to identify groups within populations in the used datasets (Fig 4A and 4B). The clonal complex (CC) CC3 and its descendants ST39, ST93, ST77, and ST53, and additional linked STs (e.g. ST15 and ST5) were clustered together. These seven STs were separated by one allele from the second group, which was composed by CC525, and its descendants ST526, ST527, and ST212 with additional linked STs (e.g. ST504 and ST311) (Fig 4B). The results did not show any pattern of differentiation in relation to their source (e.g. clinical or environmental) (S4 Fig), although seven STs were composed only by environmental isolates (e. g. ST3, ST5, ST15, ST504, ST525, ST527, and ST528) or mainly by environmental isolates, as ST77 (Fig 4B).

From six patients, two or more isolates were recovered from the same or different anatomical site at different relapse events. They were renamed as follows: patient one (P1), two isolates from CSF (L29 and L292); patient two (P2), two from blood (SG02 and SG21); (P3), one from CSF and another from blood (L50 and SG11); (P4), one from CSF, one from urine and another from blood (L58, UR01, and SG05); (P5), one from sputum and another from urine (ESC1 and UR03) and (P6), one isolate from CSF and another from blood (L69 and SG15). The multiple isolates of the patients P1, P3, P5, and P6 presented the same STs. While the patient P2 presented isolates with two different STs (ST121 and ST93) and patient P4 presented isolates with three different STs (ST212, ST526, and ST93) (Fig 1, S1 Table), indicating mixed infections.

### Susceptibility profile and virulence factors

**Fig 5:**
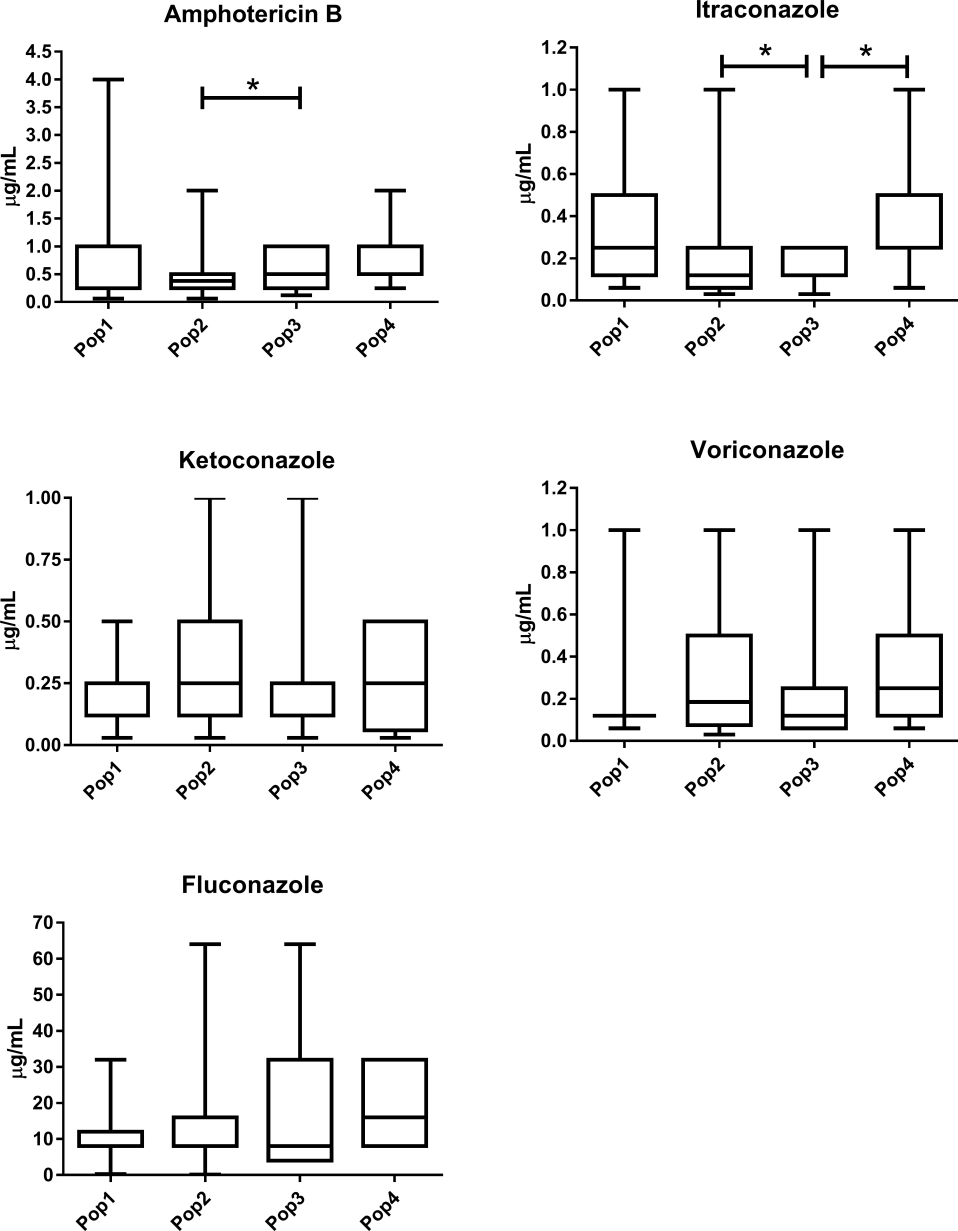
Comparison of the antifungal susceptibility patterns (μg/mL) among VNI populations of *Cryptococcus neoformans* found in this study for the antifungals tested. Statistically significant differences (p <0.05) are marked with *, Kruskal-Wallis test followed by Dunn's test. The internal horizontal lines represent the median, the bars 25–75% percentiles and the horizontal lines percentiles 10–90%. Larger statistical data available in S3 Table.

Of the 102 isolates, thirteen were resistant to the tested antifungal drugs, amongst them four exhibited resistance to amphotericin B (AMB), eight for itraconazole (ITZ), and three to fluconazole (FLZ). Resistance to FLZ was found in isolates A4M from ST527, A32 from ST525, and A73 from ST77. Resistance to AMB was found in isolates, ESC1, L201, and L499 all of them ST93, and in L403 from ST39. Resistance to ITZ was evidenced in isolate L403 from ST39 and in ESC1, L514, L543, L564, L69, and UR9 from ST93. Two isolates were resistant to both AMB and ITZ (L403 and ESC1). The susceptibility profiles were different to AMB and ITZ among populations 2 and 3 and to ITZ between population 3 and 4 (Fig 5).

Among the evaluated virulence factors, differences were found in the yeast size (**μ**m) in MOPS for Pop2 (5.43 ± 0.76) when compared to Pop3 (6.40 ± 0.64) (p <0.0001) (S5 Fig, S3 Table, and Table 3). The populations found in the study showed differences regarding to their clinical or environmental source (p<0.014), when taking the disease outcome among patients infected with ST93 compared to the other ST’s into account (p = 0.037) and differences in yeast size (μm) when cultured in capsule stimulating medium (MOPS) (<0.0001) (Table 3, S3 Table).

**Table 3:** Comparison of phenotypic characteristics found among populations.

Characteristic (unit)		Pop1^a^	Pop2	Pop3	Pop4	p
**Source** ^**c**^	Clinical	3	50	18	3	0.014*
	Environmental	3	7	1	4	
**Hemolytic Activity (Pz)** ^**d**^		0.66	0.57	0.71	0.46	0.24
**Phospholipases (Pz)** ^**d**^		0.59	0.60	0.57	0.62	0.77
**Proteases (Pz)** ^**d**^		0.93	0.9	1.0	0.93	0.24
**Gelatin Hydrolysis (Pz)** ^**d**^		0.87	0.79	0.76	0.93	0.53
**Visual Melanin Production** ^**c**^	High	3	47	18	6	0.087
	Intermediate	3	10	1	1	
**Melanin Production (480 nm)** ^**d**^		0.04	0.043	0.04	0.04	0.85
**Urease (550 nm)** ^**d**^		0.578	0.571	0.57	0.61	0.74
**Yeast size SAB (μm)** ^**d**^		6.3	5.95	6.38	6.68	0.53
**Yeast size MOPS (μm)** ^**d**^		6.1	5.43	6.40	5.78	<0.0001*
**Capsule size SAB (μm)** ^**d**^		1.04	1.18	1.02	0.99	0.097
**Capsule size MOPS (μm)** ^**d**^		3.01	3.21	2.52	2.7	0.36
**Outcome** ^**c**^	Clinical Cure	0	21	0	0	0.037*
	Death	3^b^	28	1^b^	3^b^	
**Gender** ^**c**^	Female	0	12	0	0	0.533
	Male	3	37	1	3	
**Mean Age (years)** ^**d**^		37	35.3	36	42	0.689
**Origin** ^**c**^	Uberaba	1	24	0	3	0.235
	Uberaba out	2	21	1	0	

Statistically significant differences among the groups are marked with * (p <0.05).

Legend: AB–absorbance; MOPS–Growth to evaluate the ability of capsular synthesis; SAB–Sabouraud dextrose growth.

a–The clinical and epidemiological results were not performed for one of Pop1 isolates.

b–These patients were summed to evaluate the clinical outcome in patients with ST93 and other ST’s.

c–Statistical analysis performed with the Fisher's exact test or Chi-square.

d–Statistical analysis performed with Kruskal-Wallis test applying the Dunn's post-test if necessary. Larger data are available in S3 Table.

## Discussion

During recent years several studies from around the world using MLST analysis have evaluated *C*. *neoformans* isolates from clinical and environmental sources from different regions of the world in order to get a better view of its molecular epidemiology. Most of those studies followed the ISHAM-MLST consensus scheme for *C*. *neoformans* and *C*. *gattii*, favoring data comparison and overcoming problems arising from inter-laboratory reproducibility [45–52].

The present study included 102 *C*. *neoformans* isolates, of which 91 were molecular type VNI. This results are in accordance to most reports published elsewhere, showing again that VNI is the major molecular type of *C*. *neoformans* causing human infections [47]. Of the remaining 11 isolates, two were VNIV, four VNII and five were identified as VNB.

The isolates identified as VNB by MLST in the present study had been previously identified as VNII by *URA5*-RFLP [17,18]. To evaluate this discrepancy in the molecular type identification, a review of the sequences of the restriction sites for the enzymes *Hha*I and *Sau*96I in the *URA5* locus was carried out for the major molecular types of *C*. *neoformans* (S3 Fig). This analysis confirms previous studies, which emphasize the difficulty to differentiate VNB from VNII by *URA5*-RFLP due to the presence of restriction sites at the same positions in both genotypes (S3 Fig). The discrepancy between these techniques could be attributed to insufficient resolution power of the used markers, genetic drift or recombination events [53,54]. The VNB genotype was formerly described as a group of *C*. *neoformans* serotype A, being haploid highly genetically diverse and being geographically restricted to Botswana [42]. Later, other reports have found VNB isolates in different parts of the world mainly associate with a number of tree species, reinforcing the hypothesis that it is not restricted to Africa and should be considered as a distinct molecular type [54–56]. The finding of VNB isolates presenting newly described STs may suggest that these specific ST’s are restricted to Brazil. Thus, the close relationship between VNII and VNB genotypes supports the proposition that the VNB population may be underestimated, especially when only *URA5*-RFLP is being used for molecular type identification. Several laboratories around the world have been using this technique as the main molecular tool, especially because of the costs of sequencing methods and its limited availability where the major numbers of cryptococcosis cases are present. On the other hand, as Whole Genome Sequencing (WGS) studies are becoming more accessible in high income countries, further investigations applying this technique would elucidate its global distribution [11,54]. As an example, recently genomic studies emphasized that migration of the VNB lineage between Africa and South America occurred prior to its diversification and that recombination was an important ancestral force of these isolates [44].

The remaining four (3.82%) VNII isolates were recovered from CSF (L66, LN3, LN4, and LN12). This molecular type has been described from different countries among clinical and environmental isolates and was identified by molecular techniques with frequencies ranging from 0.2% to 7.7% [46,49,52,57].

The two ST’s identified from VNIV isolates were obtained from blood cultures. The epidemiology of VGIV isolates has been less studied. The available data suggest that it is more prevalent in Europe where an average frequency of 18.3% was reported [46]. A high figure of 71% was described in Italy using serotyping [58] and recently, frequencies of 29.4% and 19% were found in Serbia [59] and France [9], respectively. In contrast, a prevalence of 1%, 0.3%, 1% and 5% was shown from Oceania, Asia, Central America/South America and North America, respectively [47]. In Brazil a VNIV prevalence of 3% has been described, including clinical [55] and environmental isolates [46,60–63].

Several studies have shown that *MATα* isolates are more virulent in animal models than *MAT****a*** isolates [64]. The predominance of mating type α in isolates of *C*. *neoformans* can partly be explained by the clonal background of the isolates or even by same-sex reproduction occurring between α mating type isolates [65]. In addition, when co-infection with both types occurs, α strains present high tropism for the CNS, which may partially explain why they are the most prevalent among clinical isolates. This fact may also explain the large number of isolates with the same ST observed in the present study, such as ST93 (57 isolates, 56%) and the ST77 (19 isolates, 18.6%) [66–68].

The *C*. *neoformans* strains herein evaluated exhibited moderate genetic variability, presenting 17 STs and a haplotype diversity of 0.653. To our knowledge, the ST’s 525, 526, 527, and 528 identified in this study are being described for the first time. All but the ST527 were isolated from the environment, and the ST525 and ST528 were recovered from soil samples obtained from the hospital neighbourhood. The pattern of the polymorphisms of these isolates revealed differences in the haplotype diversity, suggesting genetic recombination among them (Table 2). The populations presented different genetic variability demonstrated by the obtained haplotype diversity of 0.867, 0.0, 0.0, and 0.952 to Pop1, Pop2, Pop3, and Pop4, respectively (Table 2). The major haplotype diversity of Pop1 and Pop4 probably occurred because it was composed by isolates from different sequence types, whereas Pop2 presented only the ST93 and Pop3 only ST77.

The high prevalence of ST93 (56%) and ST77 (18.6%) is in line with other reports from around the word, mainly from Asia [2,48,49]. In fact, ST93 has been recovered from HIV-infected patients with high prevalence in Indonesia and India, while ST77 was mostly recovered from clinical samples from India and occasionally from France, Thailand, and Uganda [14,48,49]. The recovery of both STs from clinical and environmental sources suggests that STs have not adapted specifically to environmental or clinical sources. However, whether these clinical isolates represent acquisitions from the local environment, or are due to the patient traveling with an *in situ* latent infection is not known and requires further studies with a greater number of clinical and environmental isolates.

All but five of the STs herein identified were previously found elsewhere, despite the difference in prevalence among them. The ST5 characterized from one environmental isolate was already described with frequencies ranging from 15.8% to 77.8% from clinical isolates in Europe and Asia [49,50,69]. Other STs have been occasionally studied, such as the ST28, in Germany [69]. This fact supports the hypothesis that some STs are more adapted to different ecological niches and/or may have undergone global dispersion via bird migration among others [49].

The impact of *C*. *neoformans* mixed infections on the clinical picture and outcome of patients with cryptococcosis is not yet fully understood and seems to be unusual or less evaluated, as previously suggested [9,70]. In this study, two patients presented mixed infections (P2 and P4). Patient P2 presented two different molecular types, VNI from urine and VNIV from blood. Patient P4 presented three different STs of the same molecular type from CSF, blood and urine. At admission both patients had HIV diagnosis together with cryptococcal meningitis and died after several days on antifungal therapy. These isolates were recovered within few days, supporting the hypothesis that they simultaneously presented a mixed infection and not a reinfection by isolates genetically different. Mixed infections were described in Africa in 16.7% (4/24) by MLST [14], in Serbia 28.57% (2/7) by AFLP [59], in Cameroon 42.1% (8/19) from the same sample of CSF by PCR fingerprinting ((GACA)_4_ + (GTG)_5_) [71], in France in two reports using MLST in 18.4% (9/49) [72] and 21,5% (11/51) [9]. In accordance with these reports, mixed infection could be the result of either co-inoculation or *in vivo* evolution (microevolution) [9,73]. However, microevolution could not explain the simultaneous presence of A and D serotype isolates in the same patient, as formerly described [9,59].

Previous studies reported some associations among STs of *C*. *neoformans* with different clinical and biological characteristics, such as: survival time after cryptococcosis diagnosis [14], male gender [74], immune response, capsule and melanin production [14] and antifungal resistance [49]. Herein, differences between outcome and origin among the populations were evidenced. Sequence types others than ST93 were composed by isolates of patients who presented poor outcome (Table 3), raising the possibility that these isolates represent a high virulence phenotype associated with poor outcome. However, the relationships between the pathogen genetic diversity and clinical presentation and outcome in immunocompromised hosts are very complex and needs further studies.

The susceptibility profile of molecular types and/or among STs has been formerly evaluated for *C*. *neoformans* and *C*. *gattii* [49,63]. Herein, Pop3 showed a different profile for AMB and ITZ. Resistance to AMB and to ITZ was evident in ST93 and ST39. Higher MICs for VNI compared to VNII, and significant differences for FLZ, KTZ, and VRZ were detected (data not shown). These results are interesting although the correlation between clinical outcome and susceptibility results is still unclear.

These data remark a moderate genetic and phenotypic variability among clinical and environmental *C*. *neoformans* isolates considering the number of STs and biological features herein evaluated. Of note, mixed infections were evidenced and STs were correlated with virulence factors, antifungal susceptibility, origin and outcome. Since the number of analysed isolates in this study is still limited in some formed groups, which decreases the statistical association power, studies using additional clinical and environmental isolates from other parts of Brazil could help to determine the true correlations between genetic variability and clinical outcome, antifungal susceptibility, and virulence factors related to the isolates.

## Supporting information

S1 Table Genotypic and clinical data of the 102 *C*. *neoformans* isolates studied. (XLSX)

S2 Table Phenotypic data of the 102 *C*. *neoformans* isolate studied. (XLSX)

S3 Table Summary of statistical tests used in the study, medians and variance measures. (XLSX)

S1 Fig Number of populations used in the STRUCTURE analysis calculated according to Evanno et al., 2005. (TIF)

S2 Fig Phylogenetic analysis of Brazilian VNB isolates in context with global VNB isolates inferred by neighbour-joining analysis using the concatenated data set of the seven MLST loci (*CAP59*, *GPD1*, *LAC1*, *PLB1*, *SOD1*, *URA5*, and the IGS1 region). The analysis involved 12 *C*. *neoformans* isolates from this study and 21 VNB subgenotypes of previously published global VNB isolates (*) obtained from the MLST database (mlst.mycologylab.org). The main VNB subgenotypes can be identified by different colors. The phylogenetic tree is drawn to scale, with branch lengths measuring the number of substitutions per site. Codon positions included were 1st+2nd+3rd+Noncoding. There were a total of 3,886 positions in the final dataset. Numbers at each branch indicate bootstrap values >50% based on 1,000 replicates by each of the three algorithms which presented similar topologies. The isolates from this study are identified by sequence type number (ST), followed by isolate name and by isolation source. The control isolates are identified by ST number, followed by subgenotypes, isolate name, isolation source and citation. BEHN–bird excreta of hospital neighbourhood, ECB–excreta of captive birds raised in pet-shops, CSF—cerebrospinal fluid.
(TIF)

S3 Fig Restriction sites in the *URA5* locus and *URA5*-RFLP profiles for the enzymes *Sau*96I and *Hha*I in major molecular types of *Cryptococcus neoformans*. (A) Scheme showing the positions of restriction sites in *URA5* locus for the enzymes *Sau*96I and *Hha*I in strains of molecular types VNI, VNII, VNIV and VNB. (B) Restriction profiles of *C*. *neoformans* strains of the molecular types VNI, VNII, VNIV and VNB after digestion of the *URA5* gene with the enzymes *Hha*I and *Sau96*I on 3% agarose gel. The schemes were generated by pDRAW32 software. Legend: MW—Molecular Weight 100 bp (Invitrogen, USA).
(TIF)

S4 Fig Minimum spanning trees using the goeBURST algorithm among *Cryptococcus neoformans* isolates determined by median-joining network analysis. The size of the circles corresponds to the number of isolates within that haplotype, and the numbers between haplotypes represent the genetic distance of each haplotype, excluding the gaps. The figure shows the distribution of sequence types according to clinical, biological and geographical features.
(TIF)

S5 Fig Comparison of virulence factors and biological aspects among populations of *Cryptococcus neoformans* found in the study. Statistically significant differences (p <0.05) are marked with *, Kruskal-Wallis test followed by Dunn's test. The internal horizontal lines represent the median, the bars 25–75% percentiles and the horizontal lines percentiles 10–90%. Larger statistical data available in S3 Table.
(TIF)
