# Supplementary figures and images for: Genotypic analysis of clinical and environmental Cryptococcus neoformans isolates from Brazil reveals the presence of VNB isolates and a correlation with biological factors
Source: PLoS One. 2018 Mar 5;13(3):e0193237. doi: 10.1371/journal.pone.0193237 (PMC5837091; doi:10.1371/journal.pone.0193237)

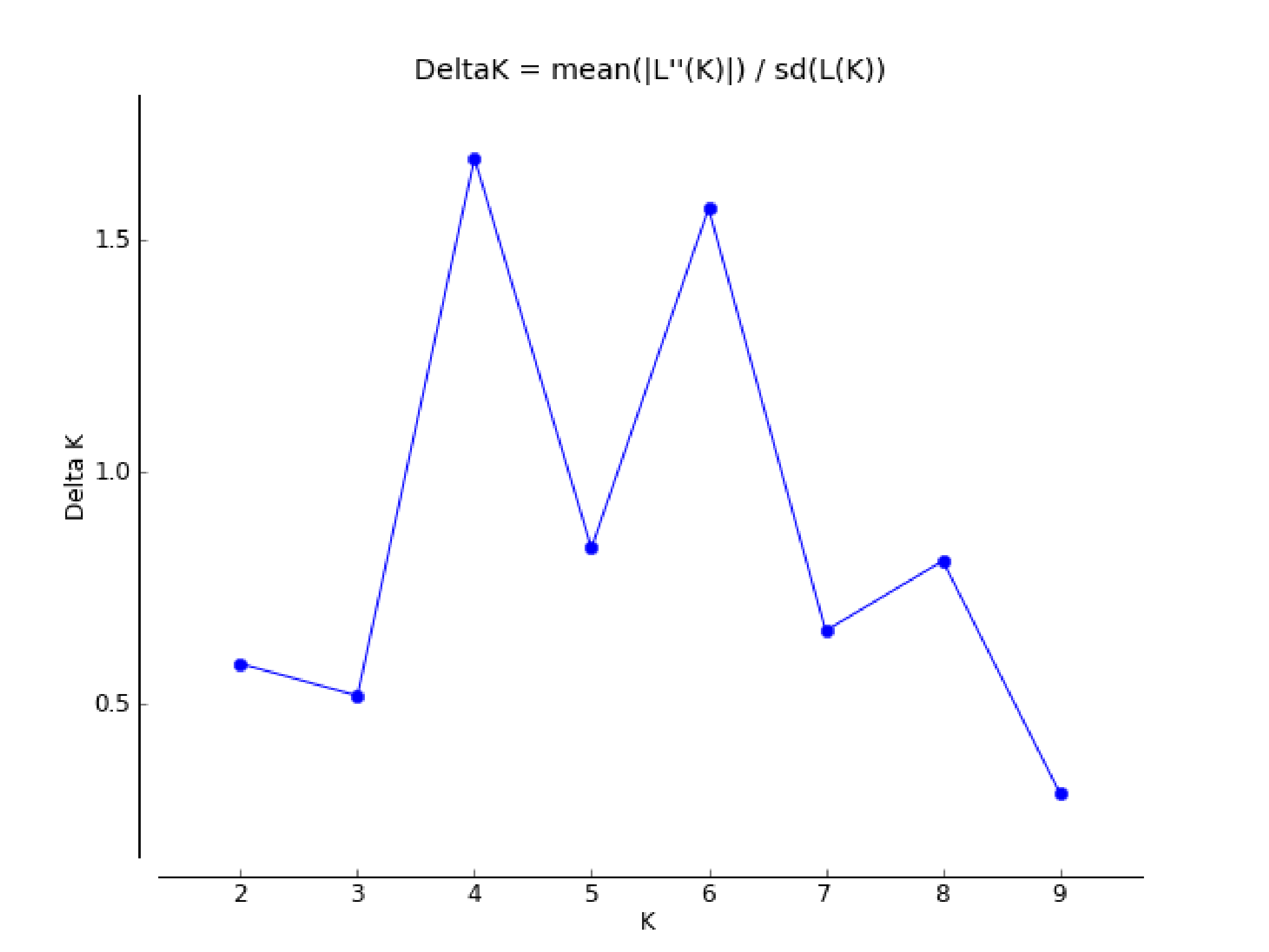

Supplement: S1 Fig — (TIF) [file pone.0193237.s004.tif]

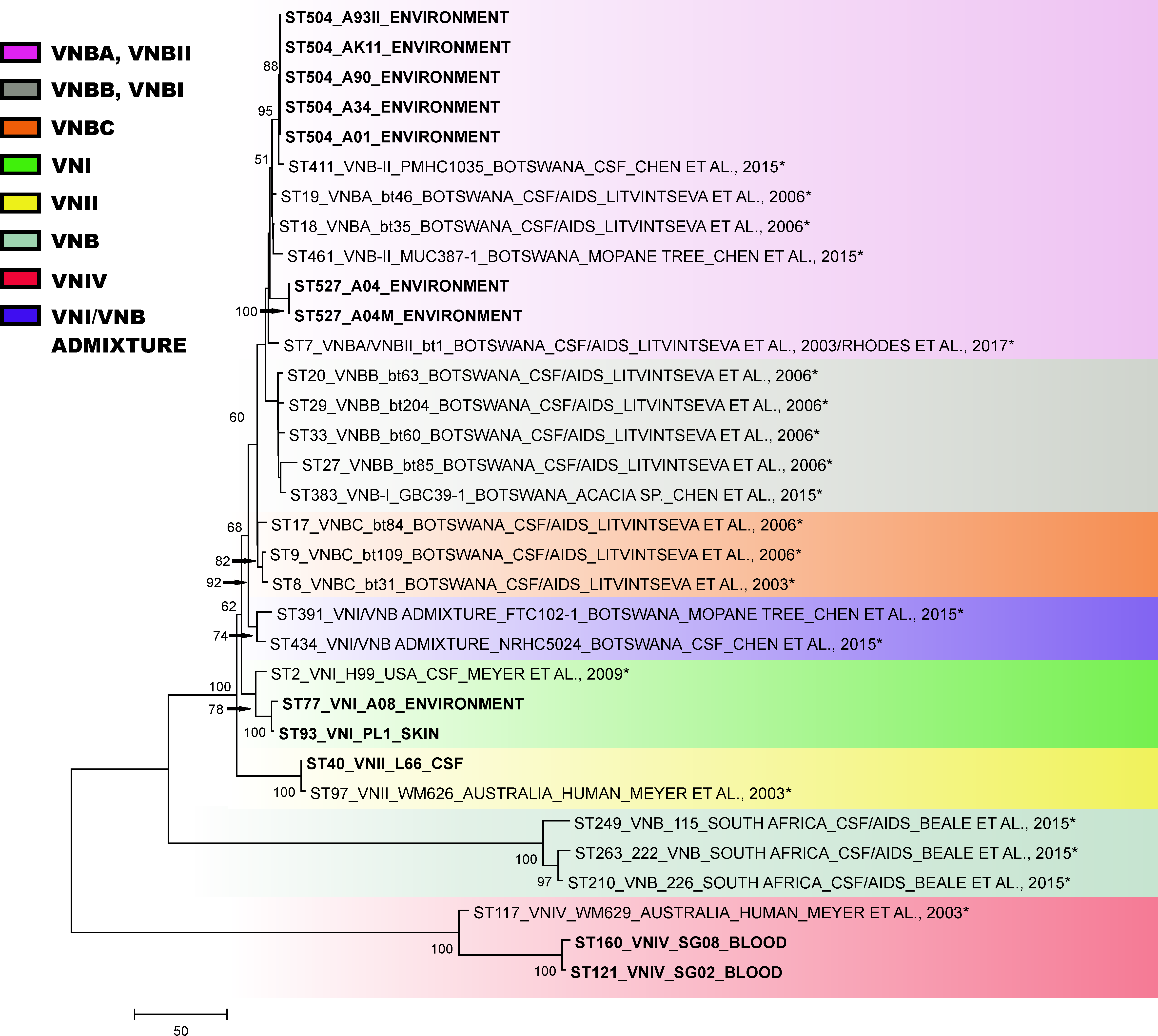

Supplement: S2 Fig — The analysis involved 12 C. neoformans isolates from this study and 21 VNB subgenotypes of previously published global VNB isolates (*) obtained from the MLST database (mlst.mycologylab.org). The main VNB subgenotypes can be identified by different colors. The phylogenetic tree is drawn to scale, with branch lengths measuring the number of substitutions per site. Codon positions included were 1st+2nd+3rd+Noncoding. There were a total of 3,886 positions in the final dataset. Numbers at each branch indicate bootstrap values >50% based on 1,000 replicates by each of the three algorithms which presented similar topologies. The isolates from this study are identified by sequence type number (ST), followed by isolate name and by isolation source. The control isolates are identified by ST number, followed by subgenotypes, isolate name, isolation source and citation. BEHN–bird excreta of hospital neighbourhood, ECB–excreta of captive birds raised in pet-shops, CSF—cerebrospinal fluid. (TIF) [file pone.0193237.s005.tif]

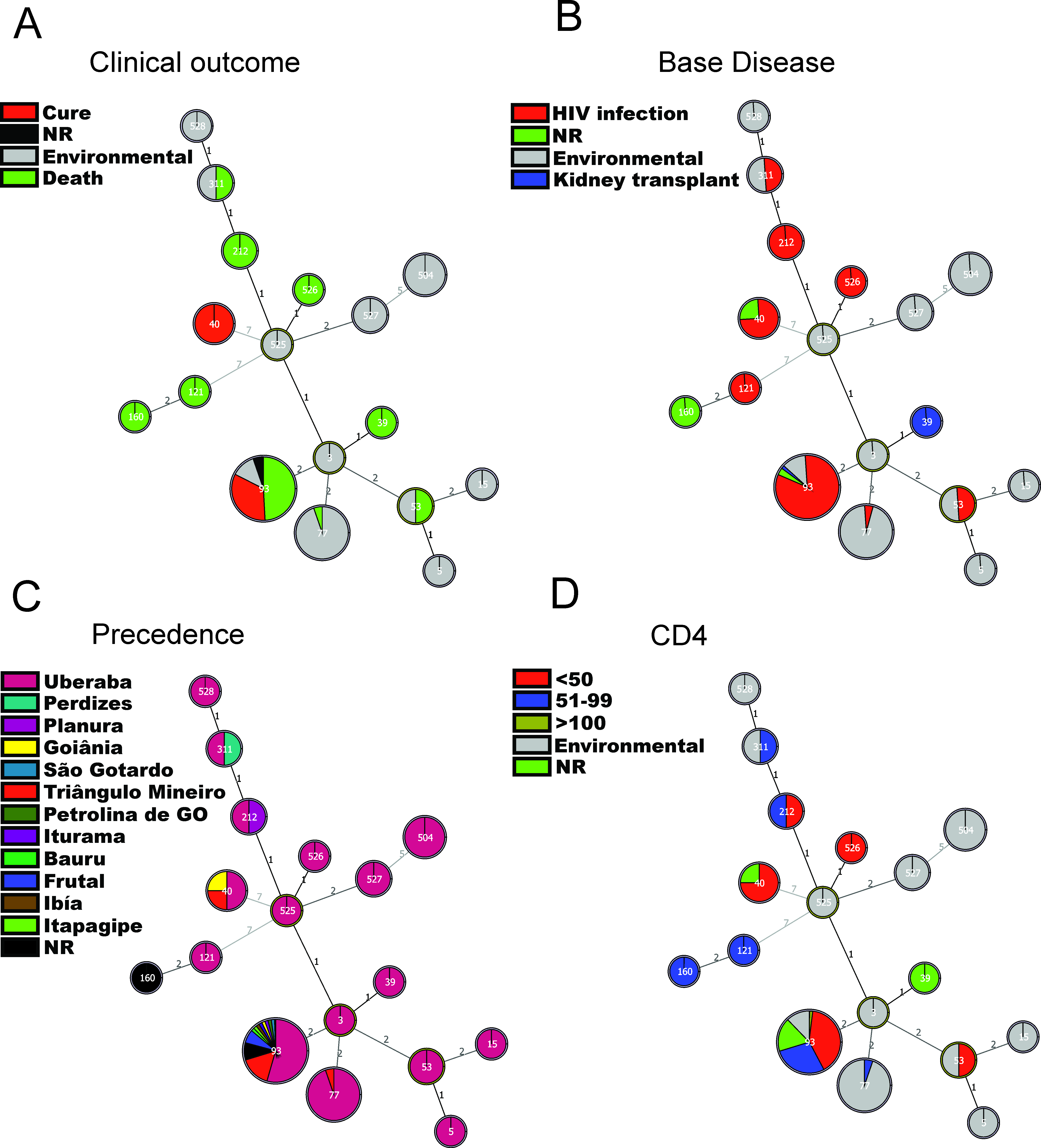

Supplement: S4 Fig — The size of the circles corresponds to the number of isolates within that haplotype, and the numbers between haplotypes represent the genetic distance of each haplotype, excluding the gaps. The figure shows the distribution of sequence types according to clinical, biological and geographical features. (TIF) [file pone.0193237.s007.tif]

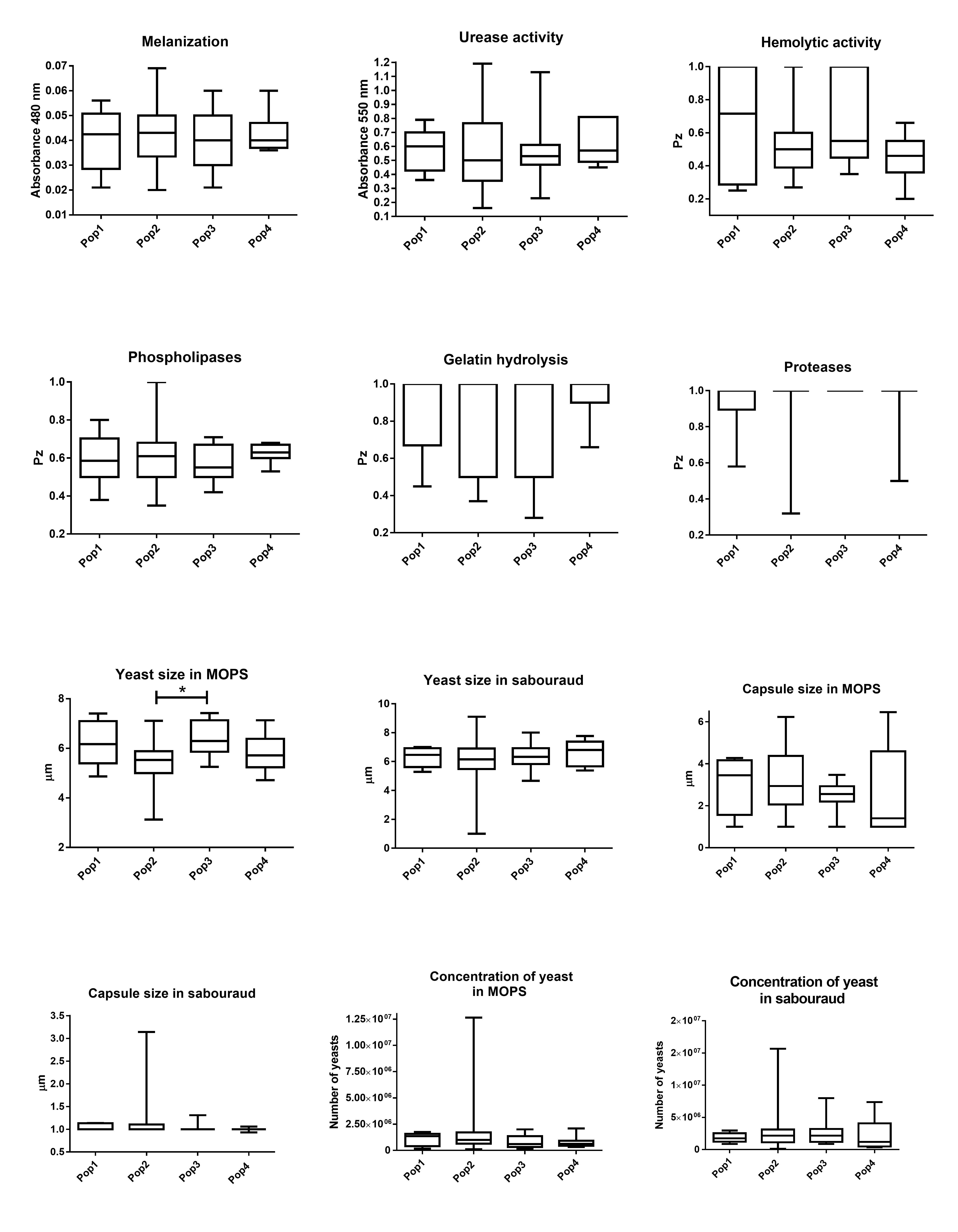

Supplement: S5 Fig — Statistically significant differences (p <0.05) are marked with *, Kruskal-Wallis test followed by Dunn's test. The internal horizontal lines represent the median, the bars 25–75% percentiles and the horizontal lines percentiles 10–90%. Larger statistical data available in S3 Table. (TIF) [file pone.0193237.s008.tif]
